# Identification of circRNA–miRNA–mRNA regulatory network in gastric cancer by analysis of microarray data

**DOI:** 10.1186/s12935-019-0905-z

**Published:** 2019-07-16

**Authors:** Yong-jun Guan, Jian-ying Ma, Wei Song

**Affiliations:** 1 Department of Hepatobiliary Surgery, Renmin Hospital of Wuhan University, Wuhan, 430060 Hubei China; 2 Department of Breast Surgery, Thyroid Surgery, Huangshi Central Hospital of Edong Healthcare Group, Hubei Polytechnic University, Huangshi, 435000 Hubei China; 3 Department of Gastroenterological Surgery II, Renmin Hospital of Wuhan University, No. 238, Jiefang Road, Wuhan, 430060 China; 4 Department of Intervention and Vascular Surgery, Affiliated Suzhou Hospital of Nanjing Medical University, Suzhou Municipal Hospital, Suzhou, 215001 Jiangsu China; 5 Department of General Surgery, Yan Da International Hospital, Langfang, 065000 Hebei China

**Keywords:** Gastric cancer, circRNA, Competitive endogenous RNA, GEO, TCGA

## Abstract

**Background:**

Evidence is increasingly indicating that circular RNAs (circRNAs) are closely involved in tumorigenesis and cancer progression. However, the function of circRNAs in gastric cancer (GC) are still unknown. Here, we aimed to determine the regulatory mechanism of circRNAs in GC.

**Methods:**

Expression profiles of circRNAs were downloaded from four Gene Expression Omnibus (GEO) microarray datasets. Expression profiles of miRNAs and mRNAs were collected from The Cancer Genome Atlas (TCGA) database. We used the robust rank aggregation method to identify differentially expressed circRNAs (DEcircRNAs) and a ceRNA network was constructed based on circRNA–miRNA pairs and miRNA–mRNA pairs. Functional and pathway enrichment analyses were performed and interactions between proteins were predicted using Cytoscape. Aa subnetwork regulatory module was built using the MCODE plugin.

**Results:**

A total of eight DEcircRNAs, 240 DEmiRNAs, and 4578 DEmRNAs were identified. The circRNA–miRNA–mRNA network was constructed based on seven circRNAs, 33 miRNAs, 69 mRNAs in GC. GO and KEGG pathway analysis indicated DEmRNAs might be associated with GC onset and progression. A PPI network was established and four hub genes (MCM4, KIF23, MCM8, and NCAPD2) were determined from the network. Then a circRNA–miRNA-hub gene subnetwork was constructed based on the four DEcircRNAs, three DEmiRNAs, and four DEmRNAs.

**Conclusions:**

Our findings provide a deeper understanding the circRNA-related competing endogenous RNA regulatory mechanism in GC pathogenesis.

## Background

Gastric cancer (GC) is one of the most common malignancies and the second leading cause of cancer-related deaths worldwide [1, 2]. Despite advances in surgical techniques and combined chemotherapy strategies, the 5-year overall survival (OS) of GC remains poor [3]. The lack of improvement in OS is largely due to a low early diagnostic rate and a high frequency of recurrence and metastasis [4, 5]. Therefore, elucidation of the molecular mechanisms underlying GC is imperative for the development of effective diagnostic and therapeutic targets.

Circular RNA (circRNA), an emerging class of non-coding RNA, has a covalently closed loop structure in which the 3′ and 5′ ends are linked in a non-collinear way by a process termed “back-splicing” [6, 7]. The lack of 5′ caps and 3′ tails makes circRNAs resistant to exonucleases and more stable than linear RNA [8]. CircRNA is structurally stable in certain tissue, time and disease specificity [9, 10]. Therefore, circRNAs have become new hotspots.

An increasing number of studies have demonstrated that circRNAs play important regulatory roles in the development of cancers. CircRNA can decrease the cytoplasmic levels of target microRNAs (miRNAs) by absorbing miRNAs and, thus, liberate mRNA transcripts that are targeted by the miRNAs. For example, Gao et al. found that has_circ_101280 was upregulated in hepatocellular carcinoma (HCC) tissues and cell lines. In their study, overexpression of hsa_circ_101280 inhibited the expression of tumor suppressor miRNA miR-375 and increased expression of the miR-124 target gene, JAK2, which leads to cancer cell proliferation. Further experiments showed that knockdown of hsa_circ_101280 inhibited growth of HCC xenografts in nude mice, which also showed downregulation of JAK2. This study demonstrated conclusively that hsa_circ_101280 interacts with JAK2 by sponging miR-1261 in HCC [11]. Similarly, circZFR was shown to interact with C8orf4 through the sponging of miR-1261 in papillary thyroid carcinoma [12].

**Fig. 1:**
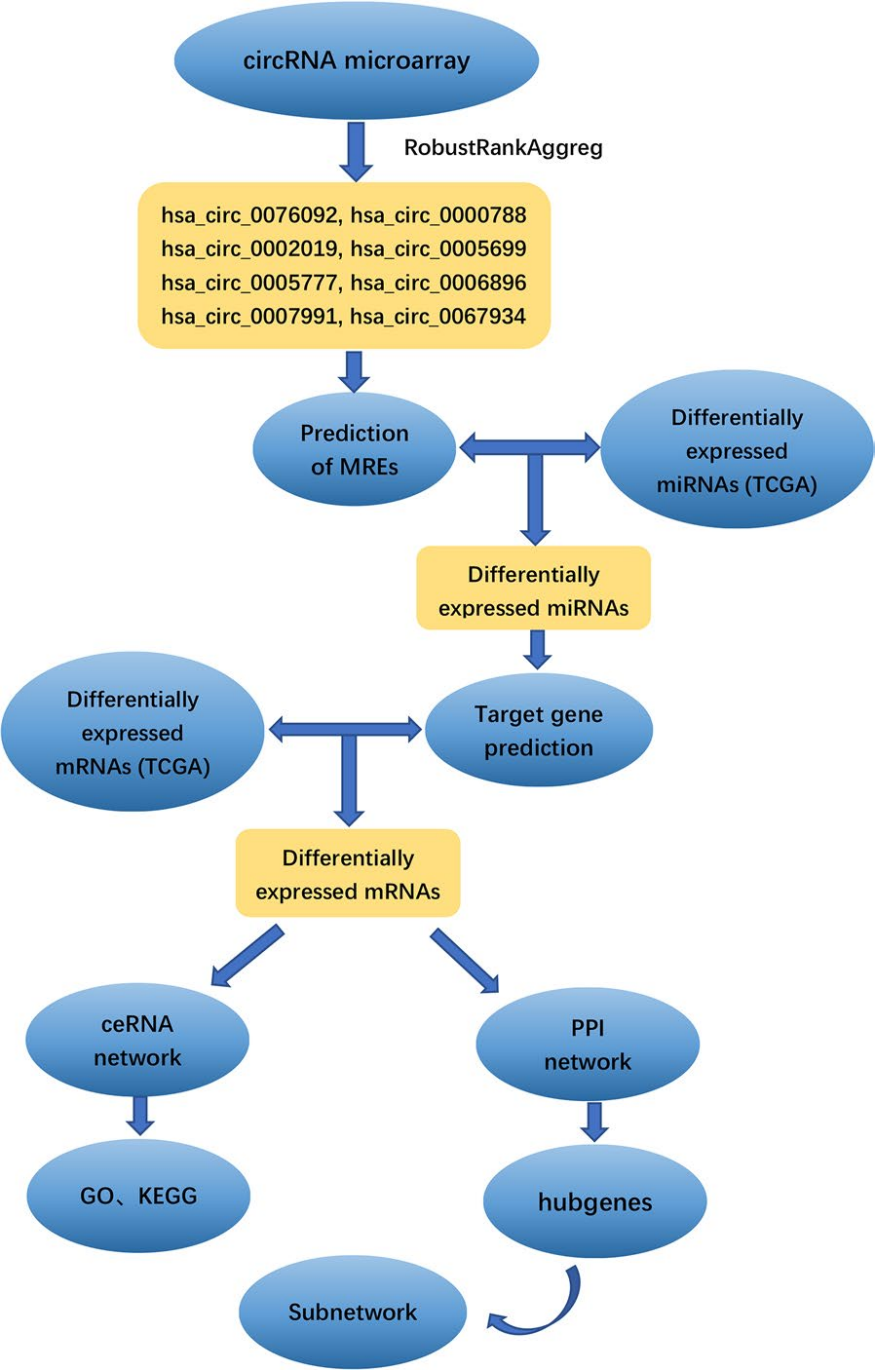
Flow chart of the approach utilized in the present study

In the current study, we collected the expression profiles of circRNAs, miRNAs, and mRNAs in GC tissues and adjacent normal gastric tissues from Gene Expression Omnibus (GEO) datasets and the TCGA database. Differentially expressed circRNAs (DECs) were identified by the RobustRankAggreg package in R. After predicting sponging of miRNAs by circRNA and miRNA target genes, we constructed a circRNA–miRNA–mRNA network. To evaluate the main functional pathways of GC, DEmRNAs of the competitive endogenous RNA (ceRNA) network were assessed by gene ontology (GO) annotation and Kyoto Encyclopedia of Genes and Genomes (KEGG) pathway analyses. We then established a protein–protein interaction (PPI) network and extracted hub genes from the PPI network. To better understand the pathogenesis of GC, a circRNA–miRNA-hub gene subnetwork regulation module was also constructed. The flowchart for this procedure is shown in Fig. 1.

## Materials and methods

### Microarray data and RNA sequencing data

The microarray data used in this study were retrieved from the GEO database. Four circRNA expression profiles were obtained from GSE78092, GSE83521, GSE93541, and GSE100170. The RNA-sequencing (RNA-seq) data were downloaded from the TCGA data portal (https://tcga-data.nci.nih.gov/tcga/). The miRNA sequencing data included 410 gastric cancer tissues and 42 adjacent normal gastric tissues, the mRNA sequencing data included 343 GC tissues and 30 adjacent normal tissues. No ethical approval nor informed consent was required in this study due to the public availability of data in the GEO and TCGA databases.

### Differentially expression analysis

Raw microarray data were normalized and log_2_-transformed. The Bioconductor Limma package was used to identify differentially expressed circRNA (DEcircRNA) in each dataset. We then integrated and ranked all of the DEGs using the robust rank aggregation method [13]. Additionally, the edgeR package was used to screen differentially expressed miRNA (DEmiRNA) and mRNA (DEmRNA) with thresholds of |log 2 (fold change [FC])| > 1 and adjusted P‐value < 0.05.

### Prediction of miRNA binding sites

The Circular RNA Interactome (CircInteractome) (https://circinteractome.nia.nih.gov/) and Cancer-Specific CircRNA (CSCD) (https://gb.whu.edu.cn/CSCD/) were used to predict miRNA binding sites (MREs). Overlapping miRNAs in the two databases were considered as potential target miRNAs of the DEcircRNAs. These target miRNAs were further screened by DEmiRNA based on The Cancer Genome Atlas (TCGA).

### Prediction of miRNA target genes

Interactions between miRNA and mRNA were predicted based on the TargetScan, miRTarBase, and miRDB databases [14–16]. Only mRNAs recognized by all three databases were considered as candidate mRNAs and intersections with the DEmRNAs were identified to screen out the DEmRNAs targeted by the DEmiRNAs.

### Construction of the ceRNA network

The circRNA–miRNA–mRNA regulatory network was constructed using a combination of circRNA–miRNA pairs and miRNA–mRNA pairs. Finally, the regulatory network was visualized using Cytoscape 3.6.1.

### Gene ontology and pathway enrichment analysis

To assess the main functional pathways of GC, DEmRNAs in the ceRNA network were assessed by GO annotation and KEGG pathway analyses with the clusterProfiler package [17] in R. A P-value of less than 0.05 was set as the cut-off criterion.

### Construction PPI network and module analysis

Based on the DEmRNAs identified, the Search Tool for the Tetrieval of Interacting Genes (STRING) database was used to construct a protein–protein interaction (PPI) network. Visualization was performed using Cytoscape 3.6.1. The Molecular Complex Detection (MCODE) app was used to screen modules of hub genes from the PPI network [18].

## Results

### Identification of DEGs in GC

**Fig. 2:**
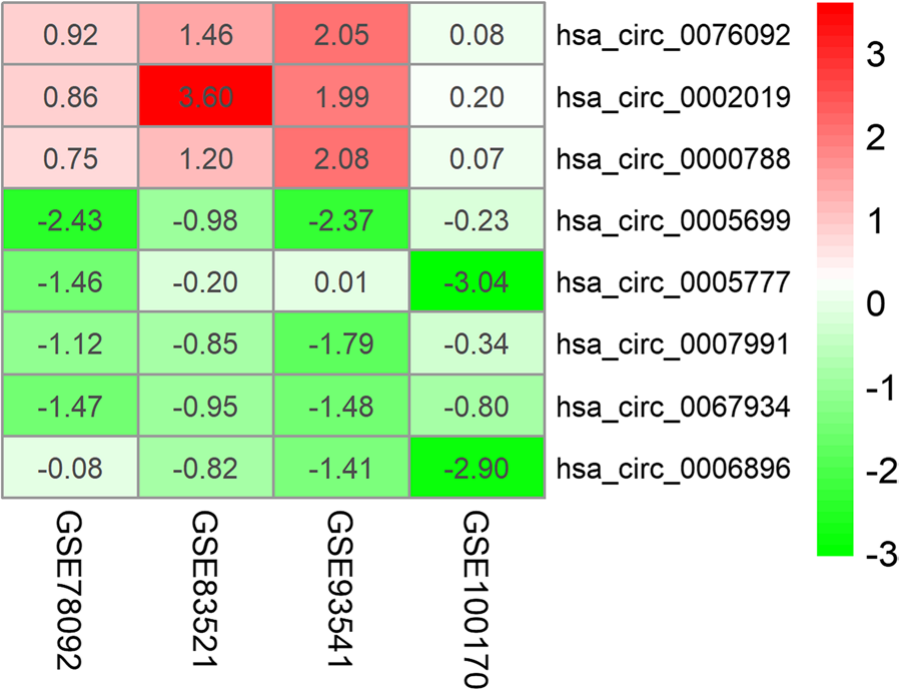
Heatmap of the eight differentially expressed circRNAs in the four microarray datasets

**Fig. 3:**
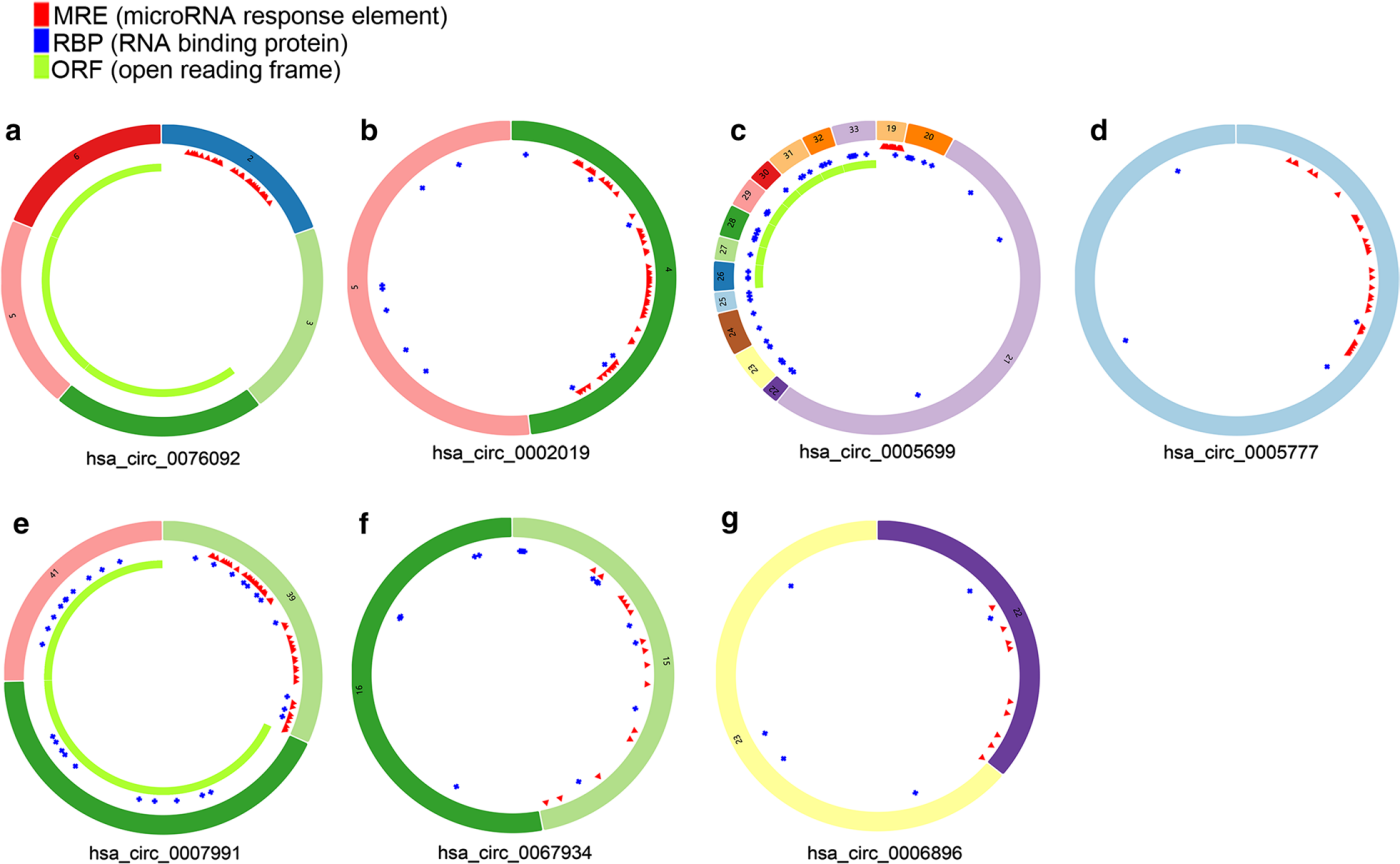
Structural patterns of the seven circRNAs: **a** hsa_circ_0076092, **b **hsa_circ_0002019, **c** hsa_circ_0005699, **d** hsa_circ_0005777, **e** hsa_circ_0007991, **f** hsa_circ_0067934, **g** hsa_circ_0006896

Expression of circRNAs in GC and control tissues was evaluated in four microarray datasets (GSE78092, GSE83521, GSE93541, and GSE100170), the basic information of which are listed in Table 1. A total of 112 DEcircRNAs, 23 upregulated and 89 downregulated, were identified in the GSE78092 dataset. A total of 73 DEcircRNAs, 43 upregulated and 30 downregulated, were identified in the GSE83521 dataset. A total of 306 DEcircRNAs, 146 upregulated genes and 160 downregulated, were identified in the GSE93541 dataset. A total of 1414 DEcircRNAs, 537 upregulated and 877 downregulated, were identified in the GSE100170 dataset. The DEcircRNAs from each of the four datasets were ranked and a total of eight DEcircRNAs, three upregulated and five downregulated, were present in the top rank (P < 0.05) (Fig. 2). The basic characteristics of the eight circRNAs are listed in Table 2. Their basic structural patterns are in shown Fig. 3. A total of 240 DEmiRNAs, 180 upregulated and 60 downregulated, and 4578 DEmRNAs, 2403 upregulated and 2176 downregulated, DEmiRNAs, were identified in the TCGA database (P < 0.05 and logFC > 1).

**Table 1:** Basic information of the 4 microarray datasets from GEO

Data source	Platform	Author	Year	Area	Sample size (T/N)	No. of circRNAs
GSE78092	GPL21485	Huang	2016	China	3/3	2902
GSE83521	GPL19978	Zhang	2017	China	6/6	3071
GSE93541	GPL19978	Guo	2017	China	3/3	1751
GSE100170	GPL23259	Wang	2017	China	5/5	88,012

**Table 2:** Basic characteristics of the eight differently expressed circRNAs

circRNA ID	Position	Genomic length	Strand	Best transcript	Gene symbol	Regulation
hsa_circ_0000788	chr17:55372279–55372525	246	+	NM_170721	MSI2	Up
hsa_circ_0002019	chr11:126142863–126143349	486	+	NR_037648	FOXRED1	Up
hsa_circ_0076092	chr6:35195356–35201078	5722	+	NM_152753	SCUBE3	Up
hsa_circ_0005699	chr16:19627435–19663412	35,977	+	NM_020314	C16orf62	Down
hsa_circ_0005777	chr5:73136304–73136585	281	+	NM_001080479	RGNEF	Down
hsa_circ_0007991	chr1:21329205–21415706	86,501	−	NM_001198801	EIF4G3	Down
hsa_circ_0067934	chr3:170013698–170015181	1483	+	NM_002740	PRKCI	Down
hsa_circ_0006896	chr8:95549330–95550574	1244	-	NM_015496	KIAA1429	Down

### Construction of the ceRNA network

**Fig. 4:**
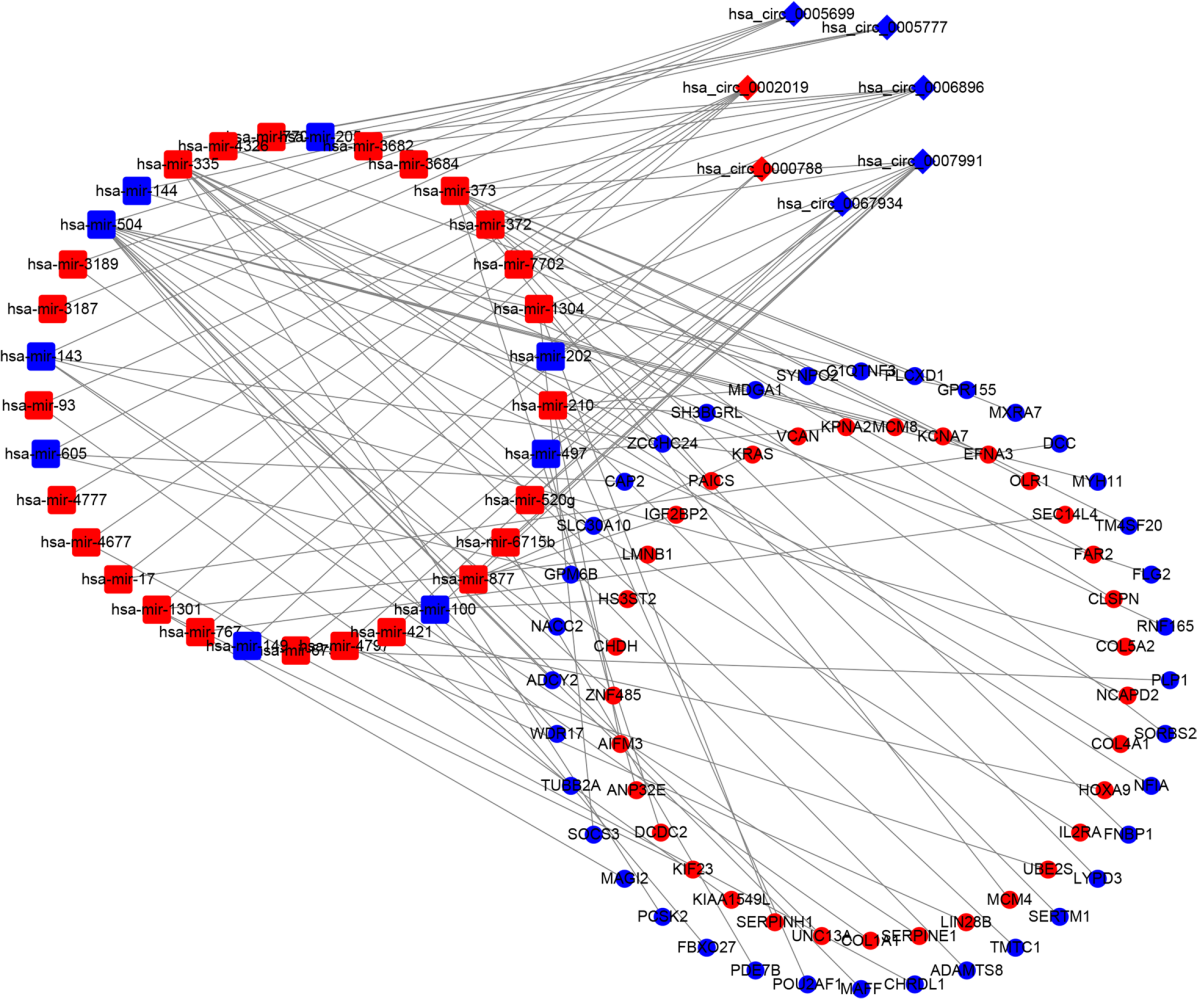
The ceRNA network of circRNA–miRNA–mRNA in GC. Diamonds indicate circRNAs, rounded rectangles indicate miRNA, and ellipses indicate mRNA. The nodes highlighted in red and blue represent up-regulation and down-regulation, respectively

To better understand the role of circRNAs and miRNAs in the ceRNA network of GC tissues, we established a circRNA–miRNA–mRNA (ceRNA) network. We retrieved data relating to the eight top-ranked DEcircRNAs identified from the microarray datasets from the CSCD and CircInteractome online databases and identified 349 pairs of interacting circRNAs and miRNAs. After intersecting with the DEmiRNAs, only 35 circRNA–miRNA pairs, including seven circRNAs and 33 DEmiRNAs, remained. We then identified mRNAs targeted by these 33 DEmiRNAs in three databases (miRDB, miRTarBase, and TargetScan). These targeted mRNAs were cross-checked against the DEmRNAs retrieved from TCGA database. These results indicated that 69 DEmRNAs were involved in the ceRNA network. Finally, we constructed a ceRNA network based on seven circRNA nodes, 33 miRNA nodes, and 69 mRNA nodes in GC (Fig. 4).

### Functional and pathway enrichment analyses

**Fig. 5:**
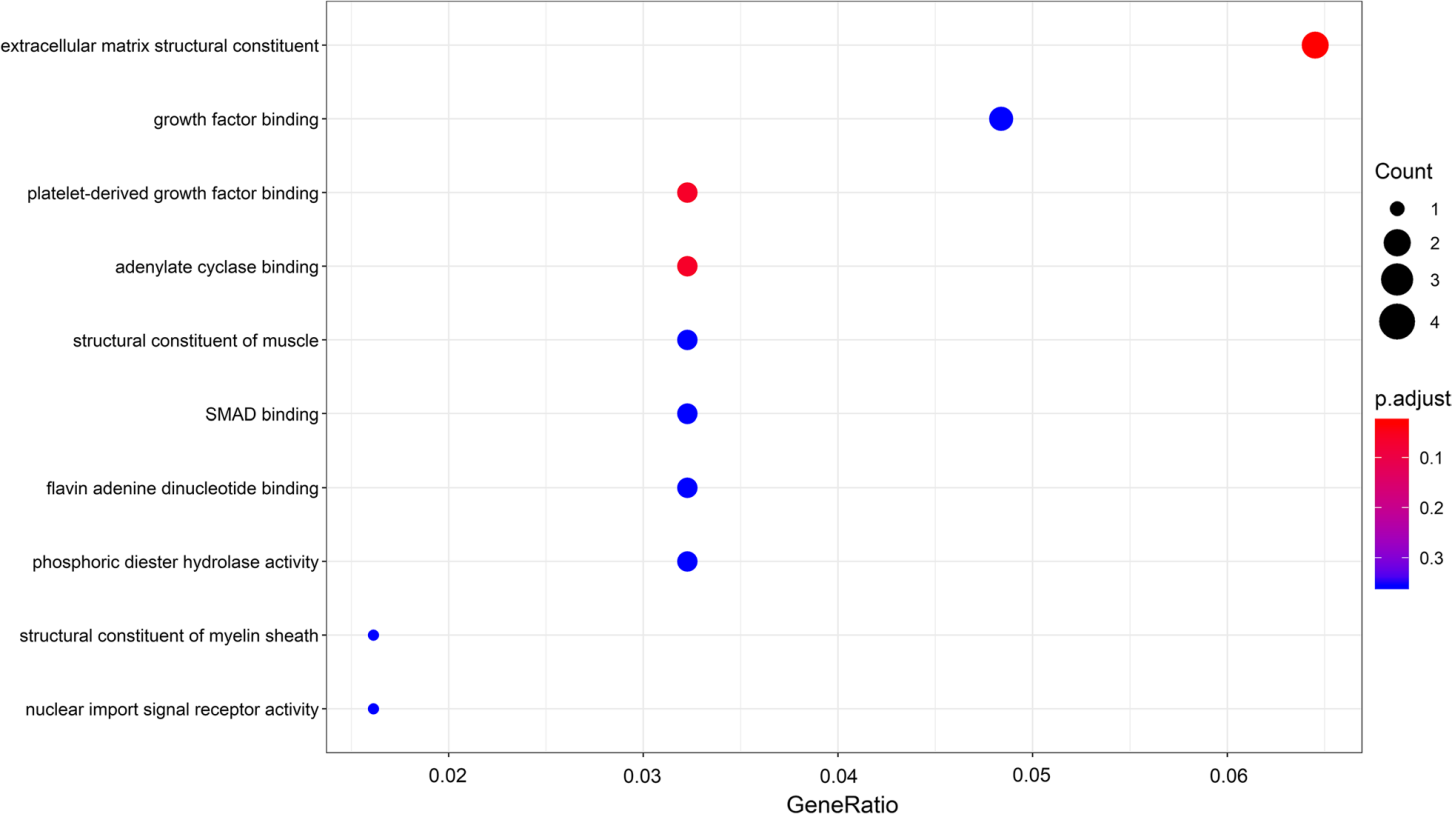
Ten most enriched GO, in terms of DEmRNAs, involved in the ceRNA network

**Fig. 6:**
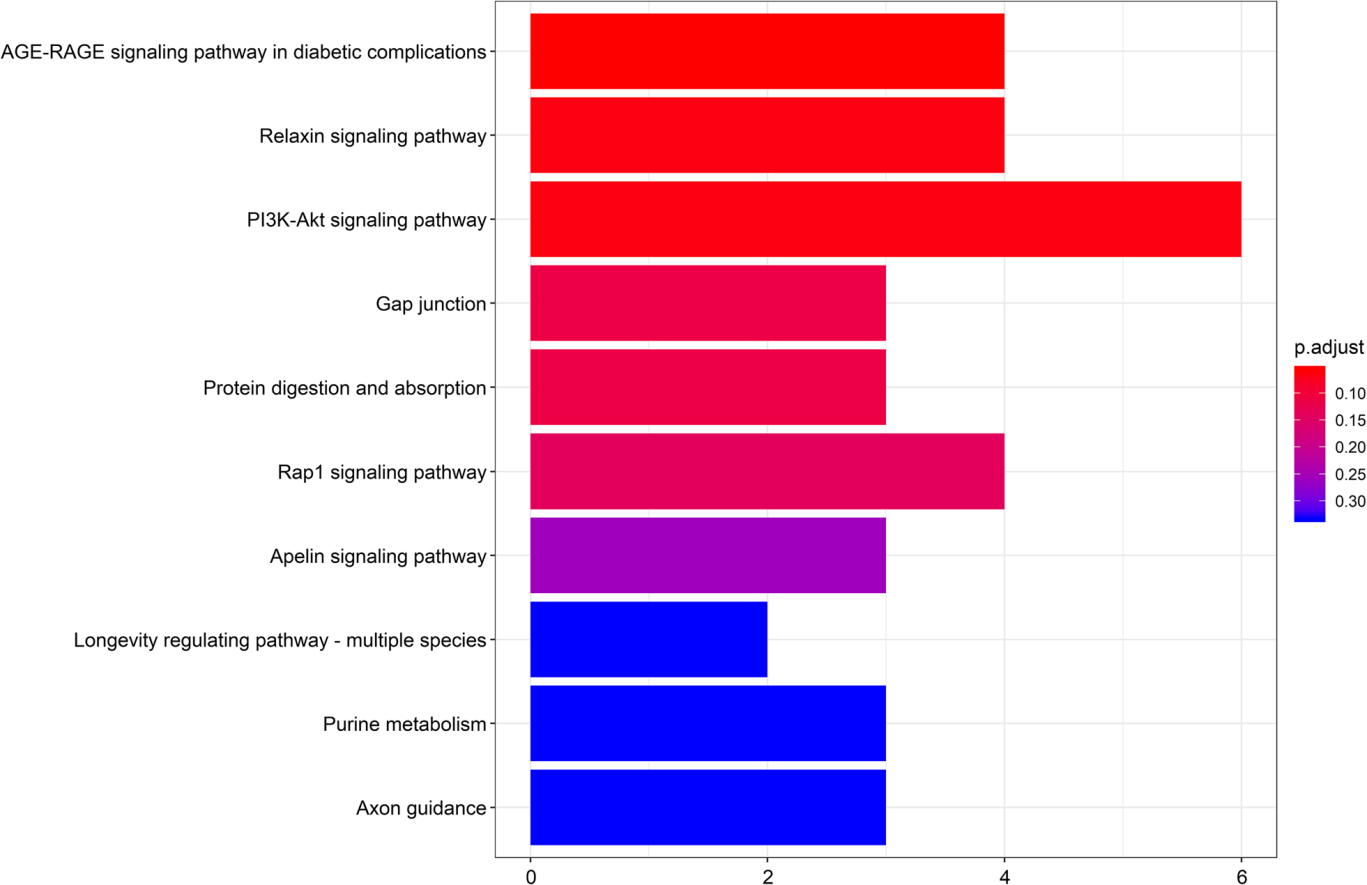
Ten most enriched KEGG pathways, in terms of DEmRNAs, involved in the ceRNA network

Gene ontology analysis revealed that the 69 DEmRNAs were enriched in 203 GO terms (P < 0.05). The top 10 enriched GO terms are shown in Fig. 5, and include “extracellular matrix structural constituent”, “platelet-derived growth factor binding”, and “adenylate cyclase binding”. The top 10 pathways associated with DEmRNAs according to KEGG analysis included “AGE-RAGE signaling pathway in diabetic complications,” “Relaxin signaling pathway,” and “PI3K-Akt signaling pathway” (Fig. 6).

### Construction of PPI network and module analysis

**Fig. 7:**
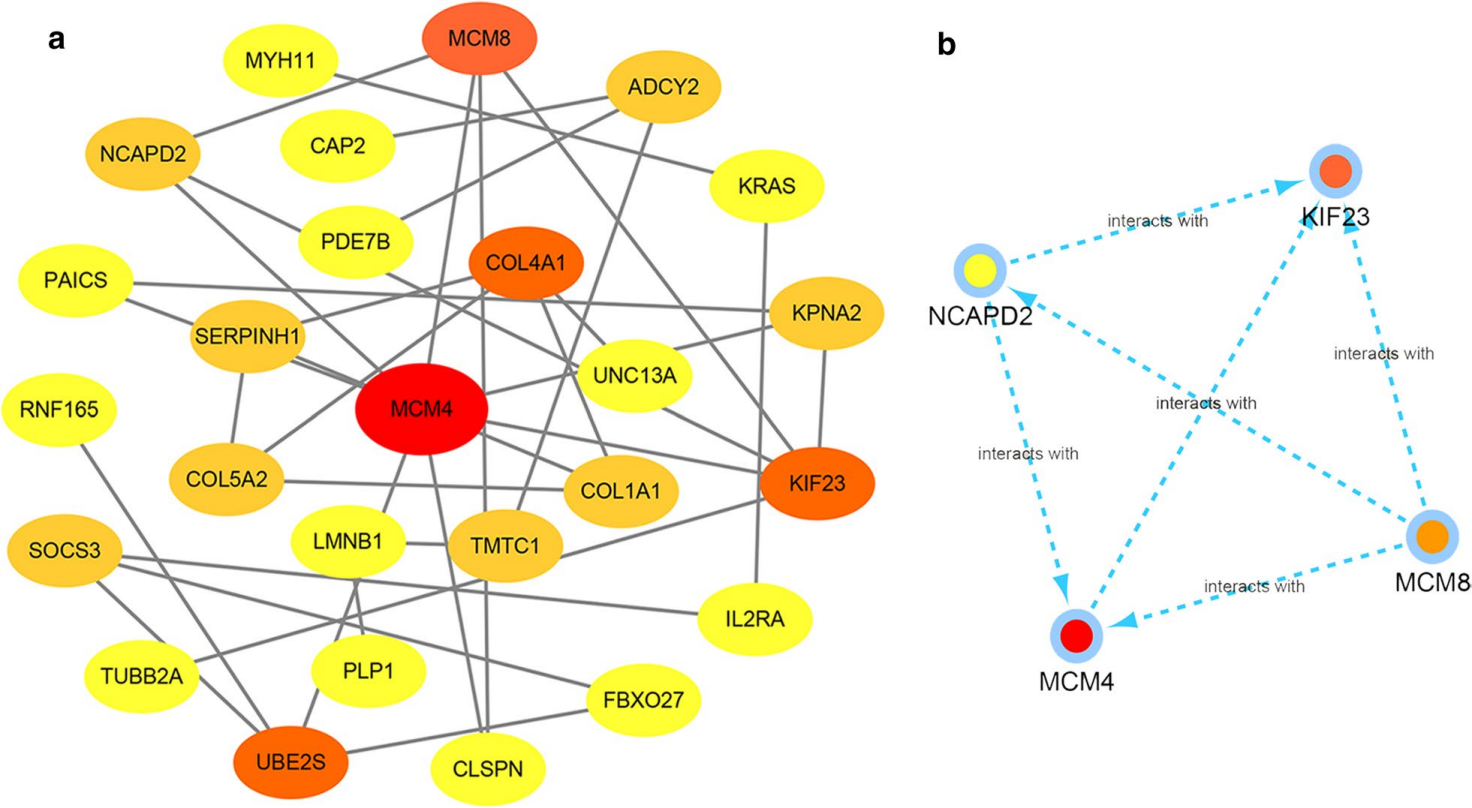
Identification of hub genes from the PPI network with the MCODE algorithm. **a** PPI network of 69 genes. **b** PPI network of four hub genes that extracted from the PPI network

In total, 26 nodes and 33 edges were mapped in the PPI network (Fig. 7a). The MCODE approach in Cytoscape was used to identify hub genes in the PPI network. With the k-core = 2, a significant module containing four nodes and six edges was identified. These highest-scoring nodes were screened as hub genes: MCM4, KIF23, MCM8, and NCAPD2 (Fig. 7b). We constructed a circRNA–miRNA-hub gene subnetwork based on five circRNA–miRNA–mRNA regulatory modules (hsa_circ_0002019/hsa-mir-1301/KIF23, hsa_circ_0005699/hsa-mir-504/MCM8, hsa_circ_0005699/hsa-mir-504/NCAPD2, hsa_circ_0006896/hsa-mir-373/MCM4, and hsa_circ_0007991/hsa-mir-373/ MCM4).

## Discussion

CircRNAs are an enigmatic type of stable, non-coding RNA that often exhibit tissue or developmental stage specific expression, although the functions of circRNAs in mammalian cells remain mostly unclear [19, 20]. The high stability of circRNAs, imparted by their cyclic structures, makes these molecules potentially valuable as novel tumor biomarkers [21, 22]. Several studies have shown that circRNAs have important influence on many complicated human diseases, including malignant tumors [23, 24]. Recently, studies have unveiled how circRNAs participate in regulation of malignant biological processes [25, 26]. Other evidence has revealed that circRNAs contain multiple MREs and can bind to miRNAs, often termed “miRNA sponges,” decreasing cytoplasmic levels of miRNAs and liberating their respective downstream target mRNAs [27–29]. However, the exact role of circRNAs in GC remains largely unknown. To identify whether circRNAs function as ceRNAs in GC, we first performed microarray data analysis to examine DEGs in GC samples and normal samples using a robust rank aggregation method. We constructed a circRNA–miRNA–mRNA regulatory network based on biological predictions and developed a model PPI network of DEmRNAs. We also constructed a circRNA–miRNA-hub gene subnetwork based on regulatory modules identified in the circRNA–miRNA–mRNA network.

Numerous studies have shown that expression of circRNA is dysregulated in GC and that this dysregulation is associated with pathogenesis and prognosis, suggesting that circRNAs could be used as tumor-associated biomarkers [30]. Fang et al. revealed that circFAT1 was downregulated in GC tissues and cell lines and was associated with OS. In vitro, overexpression of circFAT1 reduced cell proliferation, migration and invasion. That same study also found that circFAT1(e2) regulates expression of Y-box binding protein-1 (YBX1) of the nucleus by cytoplasmic sponging of miR-548g [30–32]. Similarly, high circ-SFMBT2 was observed in GC tissues and was correlated with higher stages of tumors in GC. Knockdown of circ-SFMBT2 significantly inhibited proliferation of GC cells. The authors concluded that circ-SFMBT2 participates in development and progression of GC through sponge miR-182-5p that targets CREB1 [31]. Liu [32] identified that the circular RNA-ZFR inhibited cell proliferation and promoted GC apoptosis via sponging of miR-130a/miR-107 and regulating PTEN.

In the current study, seven cicRNAs (hsa_circ_0000788, hsa_circ_0002019, hsa_circ_0005699, hsa_circ_0005777, hsa_circ_0006896, hsa_circ_0007991, and hsa_circ_0067934) were identified to be involved in the ceRNA network. One of these, hsa_circ_0067934, was identified previously by Xia et al., who analyzed 51 esophageal squamous cell carcinoma (ESCC) samples and normal samples, finding that hsa_circ_0067934 was significantly up-regulated in ESCC tissues and was associated with poor differentiation, I–II T stage, and I–II TNM stage. Knockdown of hsa_circ_0067934 in vitro by siRNA can inhibit proliferation and migration of ESCC cells and blocks cell cycle progression [33]. No relevant studies have reported involvement of hsa_circ_0000788, hsa_circ_0002019, hsa_circ_0005699, hsa_circ_0005777, hsa_circ_0006896, or hsa_circ_0007991 in cancer.

It is well known that miRNA-mediated pathways are essential to tumorigenesis; miRNAs can regulate cell proliferation, differentiation, apoptosis, and migration [34]. In the current study we identified a total of 69 DEmRNAs and 33 DEmiRNAs involved in the ceRNA network, some of which have been found as a biomarker for diagnosis and prognosis. To further identify the key circRNAs participating in the regulatory network we established a PPI network, screening four hub genes, including MCM4, MCM8, NCAPD2, and KIF23. Previous work has identified two genes, E2F1 and KIF23, that play important roles in the carcinogenesis and development of GC. It is thought that E2F1 has an important role in the cell cycle pathway by regulating MCM3, which may interact with MCM4 [35]. KIF23 is highly expressed in GC tissue, and its expression is associated with advanced TNM stage, recurrence, and poor prognosis. In vitro and in vivo experiments confirmed that inhibition of KIF23 inhibits proliferation of GC cells, leading the authors to conclude that KIF23 might serve as a potential therapeutic target for GC treatment [36]. However, there are no reports linking MCM8 and NCAPD2 with GC, nor of their association with circRNAs. Here, we identified five circRNA–miRNA-hub gene axes, indicating competitive regulatory relationships of four circRNAs with the four genes in GC. However, given that these results are based solely on bioinformatics models, further in-depth studies are critical to verifying the possible role of these four axes in GC.

## Conclusions

We screened differentially expressed circRNAs, miRNAs, and mRNAs from publicly available microarray data to construct a circRNA-associated ceRNA network. The circRNA–miRNA-hub genes regulatory subnetwork uncovered four important circRNAs that might be involved in carcinogenesis, providing new insight into the pathogenesis of GC and suggesting potential therapeutic targets that warrant further investigation.

## Data Availability

The datasets used and/or analyzed during the current study are available from the corresponding author upon reasonable request.
